# Pachydermoperiostosis and Work Restrictions: A Case Report

**DOI:** 10.7759/cureus.36675

**Published:** 2023-03-25

**Authors:** Mahdi Chinichian, Omid Asghari, Niloofar Safaie, Negin Kassiri

**Affiliations:** 1 Department of Occupational Medicine, School of Medicine, Iran University of Medical Sciences, Tehran, IRN

**Keywords:** work restrictions, worker, workplace, acromegaly, pachydermoperiostosis

## Abstract

Pachydermoperiostosis (PDP) is a rare disease that mimics the clinical and radiographical manifestations of acromegaly. Therefore, it should be considered as one of the differential diagnoses in the evaluation of acromegalic patients. In this study, we discussed a case of PDP in a 24-year-old simple worker working in a food industry factory and reviewed the work restrictions caused by the complications of the disease.

## Introduction

Pachydermoperiostosis (PDP) is a rare disease with skin and musculoskeletal manifestations, which was first described in 1868 by Friedreich [1]. This disorder is often seen in males. The diagnosis of the disease is usually based on clinical manifestations and can be confirmed by performing genetic tests. PDP accounts for about 5% of all cases of hypertrophic osteoarthropathy [2]. The demonstrative criteria for PDP incorporate major criteria comprising of pachyderma, periostosis, and finger clubbing, as well as minor criteria counting hyperhidrosis, arthralgia, gastric ulcer, cutis verticis gyrata, blepharoptosis, joint effusion, column-like legs, edema, seborrhea, acne, and flushing [3,4]. In this case report, we examined a rare case of a 24-year-old man with PDP and described the job restrictions caused by the clinical manifestations of his disease.

## Case presentation

**Figure 1:**
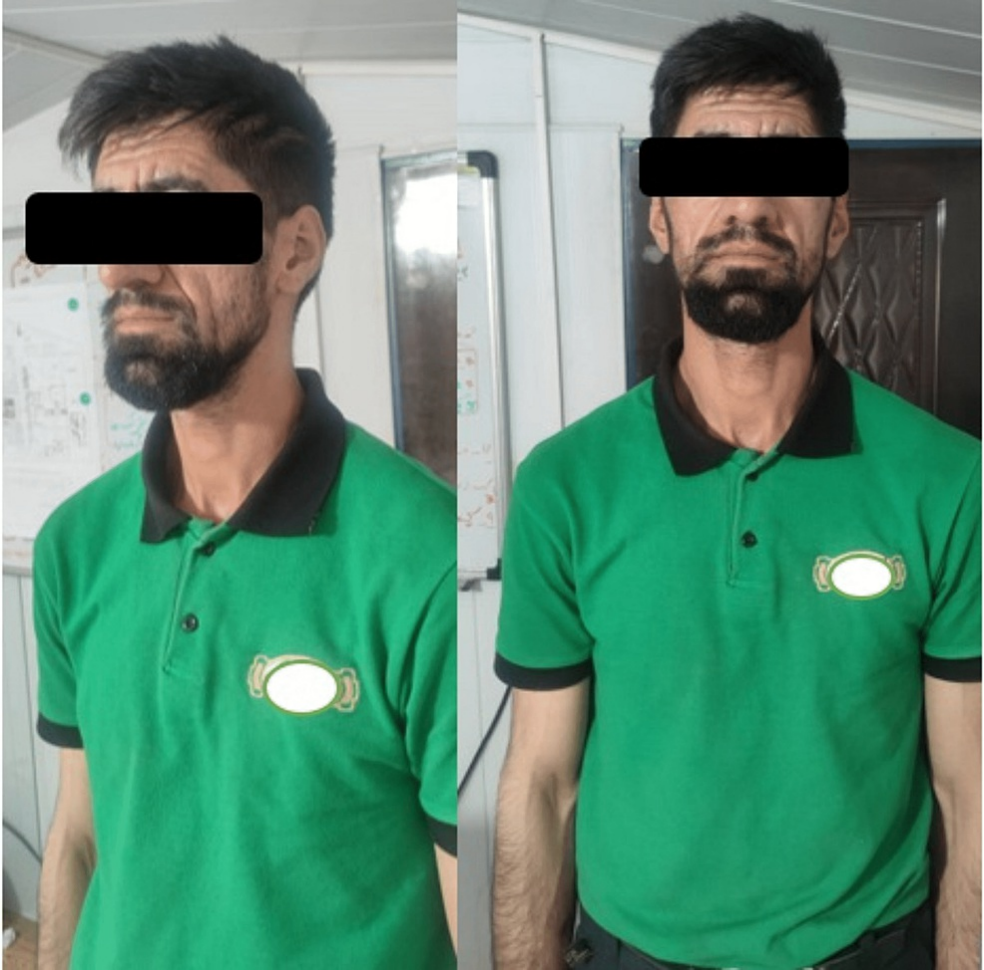
Coarseness of facial features.

**Figure 2:**
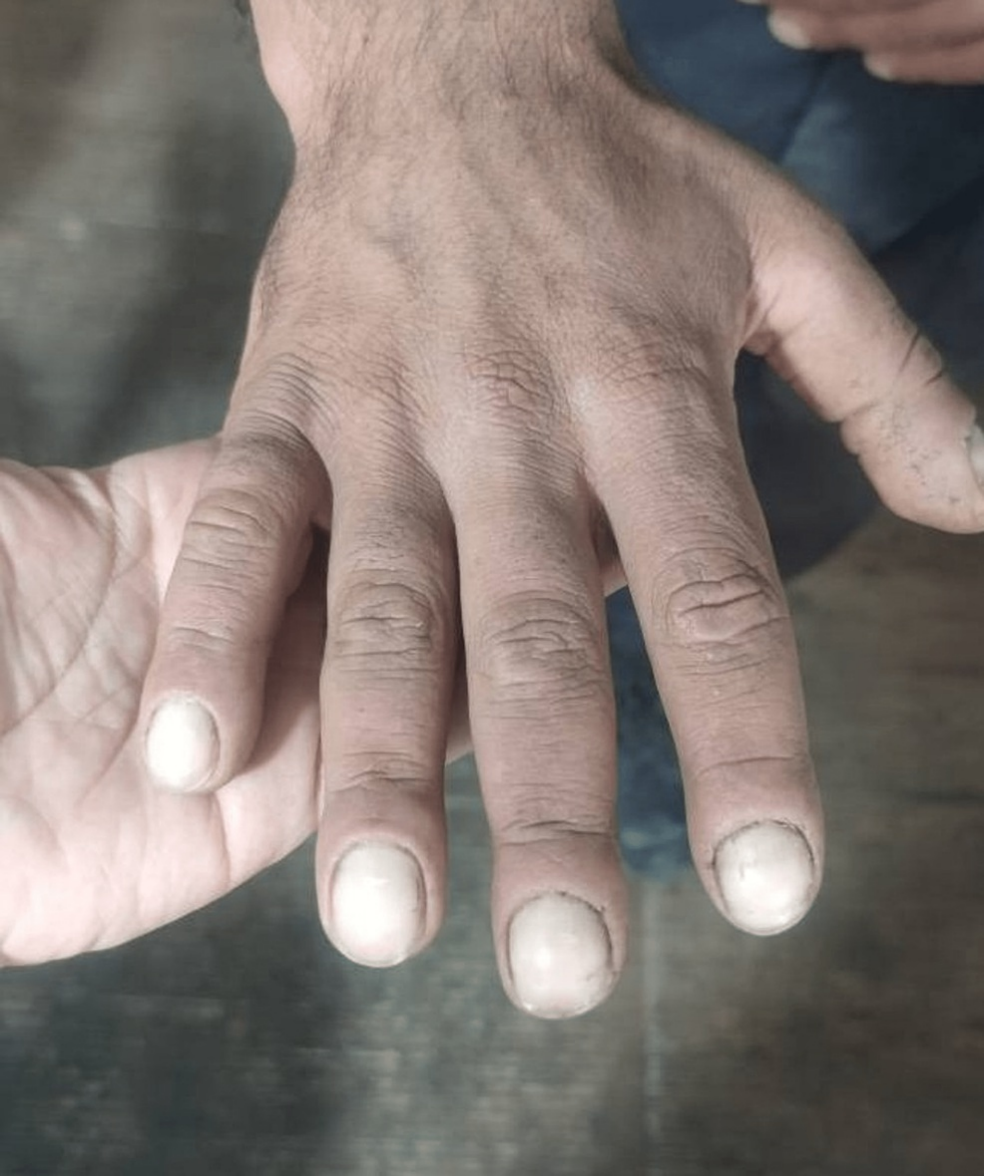
Clubbing in hand's nails.

**Figure 3:**
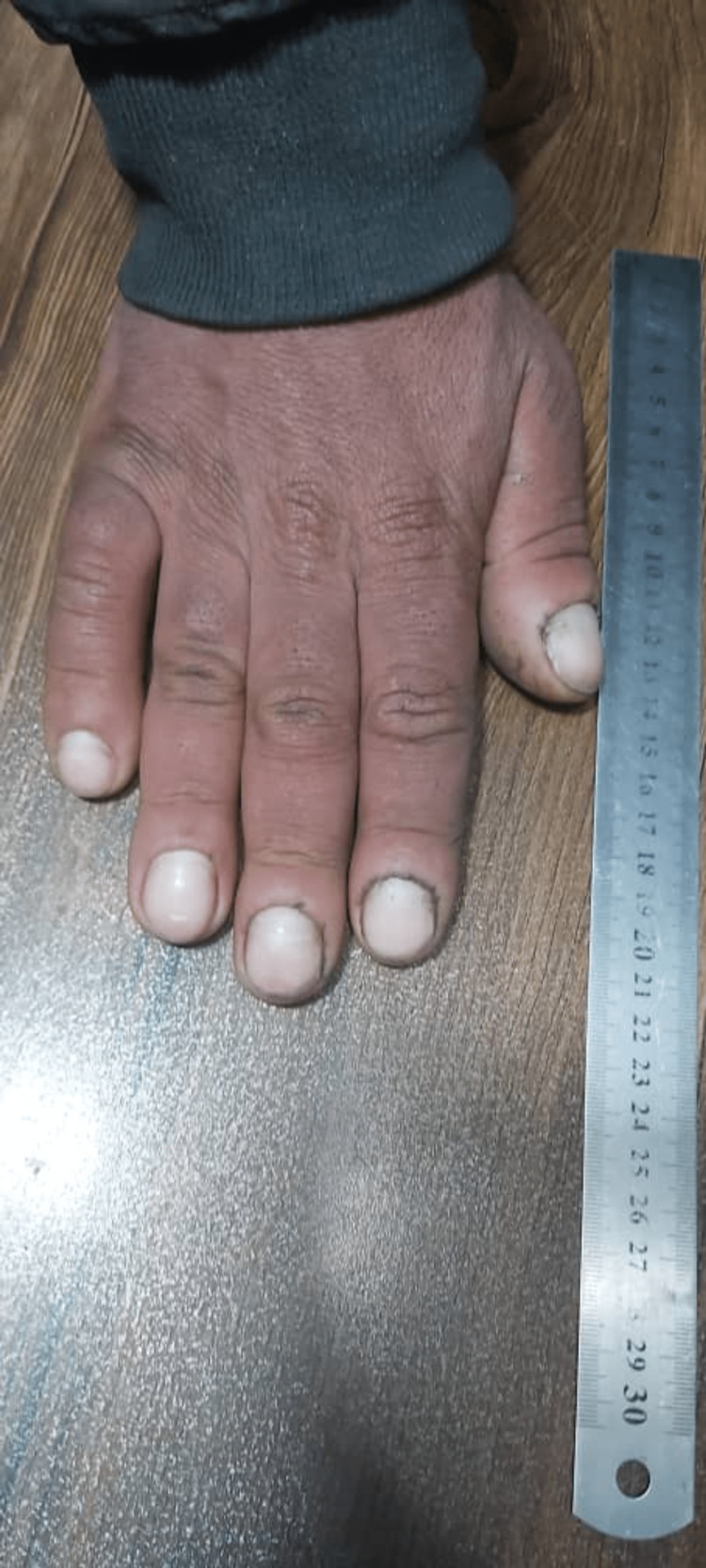
Large hand size in a male adult with pachydermoperiostosis.

**Figure 4:**
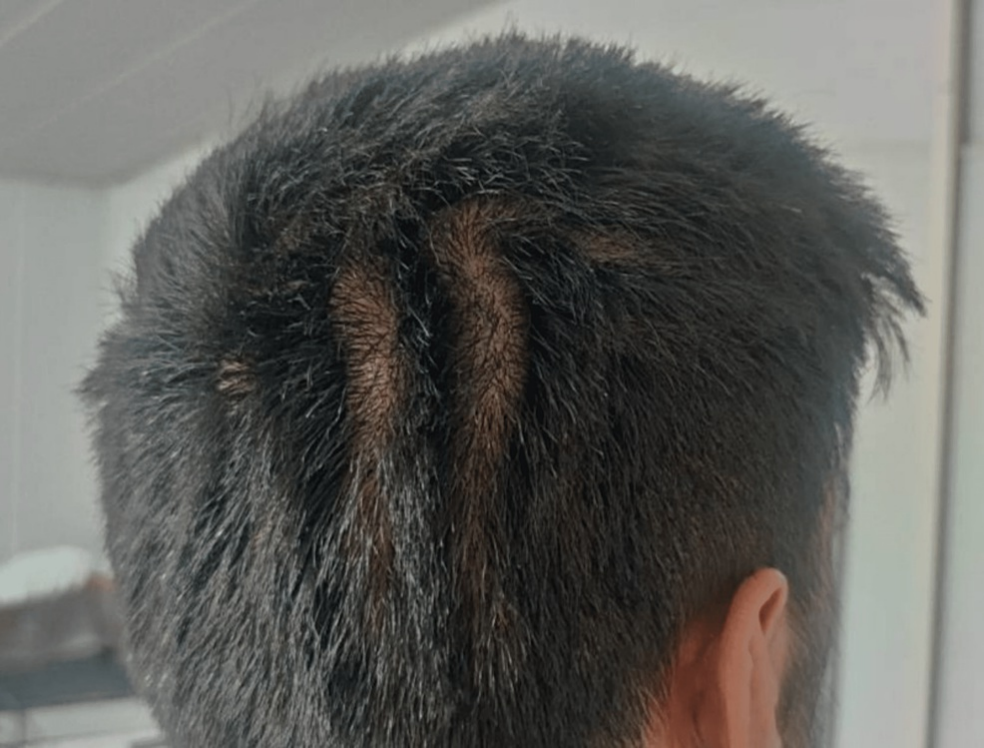
Cutis verticis gyrata in a patient with pachydermoperiostosis.

The patient was a 24-year-old male working in a large food industry factory as a simple worker for three years. In his job, he was exposed to dust, cleansers, and ergonomic hazards (including awkward postures and heavy lifting was mentioned). He had no secondary or previous job. He did not have any complaints during his previous visits and last year's medical documents do not contain any records. In the periodic occupational health evaluations, occupational medicine specialist noticed the coarseness of facial features, clubbing in nails, and large hands (Figures 1-3). Other findings were grooves in scalp skin (cutis verticis gyrata) and oily skin, furrowing in the forehead and cheeks (Figure 4).

The patient did not complain of any specific clinical symptoms. The occupational medicine specialist referred him to an endocrinologist for suspected acromegaly. The endocrinologist was suspicious of acromegaly and performed hormonal tests to confirm the diagnosis. The normal growth hormone level (GH) was reported, which was detrimental to the diagnosis of acromegaly. The hormonal tests were done by the endocrinologist two times, and the results of the second time (confirmatory test) are shown in Table 1.

**Table 1:** Laboratory test results in a patient with pachydermoperiostosis.

Hormonal markers	Values	Normal range
Insulin-like growth factor 1 (IGF-1)	122.9	138-363
Cortisol	14.1	5-25
luteinizing hormone (LH)	8.9	1.1-25
Testosterone	4.83	2.4-8.7
Prolactin	15.5	3.4-19.4
Thyroid-stimulating hormone (TSH)	1.5	0.3-5
Free thyroxin 4 (FT4)	1.1	0.7-1.8
Adrenocorticotropic hormone (ACTH)	16.5	7.2-63.3
Growth hormone (base)	1.1	Up to 4

Due to normal GH and insulin-like growth factor 1 (IGF-1) levels, acromegaly was excluded. According to the clinical symptoms and the results of hormonal tests, the diagnosis of PDP was placed on the top of differential diagnoses. In order to make a definite diagnosis genetic tests are needed, but they were not performed on this person because the cost of doing them in our country was high and the patient could not afford them. In pituitary magnetic resonance imaging (MRI), a 14x12 mm lesion in sella turcica with compression effect on the pituitary gland with deviation towards the right side was observed. The mass was not functional. Due to the involvement of the sella region, perimetry with red light was performed on the patient, and relative temporal hemianopia was reported. Fundoscopy of both eyes was normal.

## Discussion

In a patient with acromegaloid appearance, a wide range of differential diagnoses, such as acromegaly, pseudoacromegaly, Marfan’s syndrome, and rare conditions, such as PDP, are proposed. When acromegaly is suspected in a patient, the first step is performing biochemical testing to confirm the clinical diagnosis, followed by radiological testing to determine the cause of excess GH secretion. In more than 95% of cases, the cause is a growing adenoma of the pituitary gland. The first step is to measure insulin-like growth factor 1 (IGF1) levels in serum. A normal serum IGF1 concentration is strong evidence that the patient is not the case of acromegaly. Serum GH should be measured after oral glucose administration if serum IGF1 concentration is high or equivocal. Inadequate suppression of GH after glucose challenge confirms the diagnosis of acromegaly. Once the presence of excessive GH secretion is confirmed, the next step is magnetic resonance imaging (MRI) of the pituitary [5]. In this patient, since the GH and IGF levels were in the normal range, taking into account the clinical symptoms, PDP was finally diagnosed.

PDP also called hypertrophic osteoarthropathy, Rosenfeld-Kloepfer syndrome, and Touraine-Solente-Gole syndrome is a rare disease characterized by three main features - clubbing, skin thickening (pachyderma), and increased sweating (hyperhidrosis) [6,7]. This disease usually starts in childhood and adolescence, around puberty, and gradually progresses over 10 years. This disease is associated with the painful formation of new bones, congenital heart diseases, and delay in the closure of fontanelles. Patients have rough facial features and oily thick skin. Swollen and painful joints, ptosis, seborrheic dermatitis, long eyelashes, and occasional diarrhea are other symptoms of this syndrome. People may show a variety of symptoms, but serious symptoms are generally more common in men than women. In most cases, a diagnosis can be made by examining clinical symptoms [8]. This disease can be hereditary with an autosomal dominant or recessive pattern. Non-genetic forms of this disease have also been reported. Its prevalence in men is 7:1 compared to women. Symptoms of PDP may be mistaken for osteoarthritis, psoriatic arthritis, rheumatoid arthritis, and thyroid acropachy. Treatment is usually supportive. Patients can use non-steroidal anti-inflammatory drugs, colchicine, and corticosteroids to relieve joint pain. Plastic surgery can also be used to improve facial features [9,10].

In this study, we investigated a 24-year-old man with PDP. First of all, the large size of the hands of this patient attracted the attention of the occupational medicine specialist. Therefore, our advice to occupational health specialists is not to neglect the appearance examination of patients. PDP is one of the differential diagnoses of more common diseases, such as acromegaly and pituitary gland masses, which may not be considered due to very low prevalence. Occupational medicine specialists are faced with limitations and restrictions when determining the fitness for work. The limitation is raised when a person is unable to do something due to illness or its treatment complications. Restriction is defined as when a person is able to do something but is prohibited from doing it due to the possibility of harming himself or others. This disease does not have a specific effect on the patient's job, but it can be associated with symptoms such as general weakness, fatigue, and muscle pain, and may lead to a decrease in productivity. This patient had macroadenoma of the pituitary gland which cause the limitation in the field of vision, as relative temporal hemianopia and it leads to a decrease in the person's vision towards the side edges of the field of vision, so to maintain safety, this person was restricted from working in the traffic area of ​​forklifts and heavy machinery. Note that in the field of occupational medicine, the most important visual field defect is inferior hemianopia. If this defect occurs, the person will not be able to see the near fieldwork area completely and the possibility of errors and occupational accidents will increase. Another complication of this disease is excessive sweating, which increases the possibility of contact dermatitis if the hands are involved. It also increases the risk of surface rust in jobs that deal with metals. These patients are at risk of severe kyphosis, neurological manifestation, and restricted motion, so it is recommended to pay attention to the mentioned points in the periodical examinations of occupational health.

## Conclusions

PDP is an important, rare differential diagnosis of acromegaly which may mimic its clinical symptoms, but can be differentiated by performing additional laboratory tests. This disease may lead to complications, such as general weakness, fatigue, muscle pain, reduced field of vision, increased sweating, and dermatitis caused by it. In case of the above complications, occupational restrictions will be applied to the patient.
